# Cost utility analysis of Remdesivir and Dexamethasone treatment for hospitalised COVID-19 patients - a hypothetical study

**DOI:** 10.1186/s12913-021-06998-w

**Published:** 2021-09-18

**Authors:** Andrea Carta, Claudio Conversano

**Affiliations:** Department of Business and Economics, University of Cagliari, Viale S. Ignazio 17, Cagliari, 09123 Italy

**Keywords:** Sars-Cov-2, Covid-19, Remdesivir, Dexamethasone, Cost Utility, Cost Effectiveness, pharmacoeconomics, Decision tree

## Abstract

**Background:**

Sars-Cov-2 is a novel corona virus associated with significant morbidity and mortality. Remdesivir and Dexamethasone are two treatments that have shown to be effective against the Sars-Cov-2 associated disease. However, a cost-effectiveness analysis of the two treatments is still lacking.

**Objective:**

The cost-utility of Remdesivir, Dexamethasone and a simultaneous use of the two drugs with respect to standard of care for treatment Covid-19 hospitalized patients is evaluated, together with the effect of Remdesivir compared to the base model but based on alernative assumptions.

**Methods:**

A decision tree for an hypothetical cohort of Covid-19 hospitalized patients, from an health care perspective and a one year horizon is specified. Efficacy data are retrieved from a literature review of clinical trials, whilst costs and utility are obtained from other published studies.

**Results:**

Remdesivir, if health care costs are related to the days of hospitalization, is a cost saving strategy. Dexamethasone is cost effective with an ICER of <DOLLAR/>5208/QALY, and the concurrent use of Remdesivir and Dexamethasone is the most favorable strategy for higher level of willingness to pay thresholds. Moreover, if Remdesivir has a positive effect on mortality the utility is three times higher respect to base case. Whereas, if health care costs are not related to the length of patient hospitalization Remdesivir has an ICER respect to standard of care of <DOLLAR/>384412.8/QALY gained, which is not cost effective. We also find that Dexaamethasone is cost effective respect to standard care if we compute the cost for live saved with an ICER of <DOLLAR/>313.79 for life saved. The uncertainty of the model parameters is also tested through both a one-way deterministic sensitivity analysis and a probabilistic sensitivity analysis.

**Conclusion:**

We find that the use of Remdesivir and/or Dexamethasone is effective from an economic standpoint.

## Background

COrona VIrus Disease 2019 (COVID-19), caused by severe acute respiratory syndrome coronavirus 2 (SARS-CoV-2), was first reported in China in December 2019 [1], however phylogenetic estimates support that the Covid-19 pandemic started as far as October 2019 [2]. As of December 2020 the novel corona virus has caused significant mortality and morbidity, as there have been more than 70 million documented cases and more than one million deaths [3], both are probably globally under counted [4, 5]. Consequently there is a great interest in finding potential treatments for the respiratory disease.

Although there was a great enthusiasm for many possible candidate drugs such hydroxycloroquine or lopiravir [6, 7], only two therapeutic agents have shown benefits in robust Randomized Control Trials (RCT): Remdesivir and Dexamethasone. Remdesivir is a 1’-cyano-substituted adenosine nucleotide analogue prodrug developed for the treatment of Ebola virus disease [8], but has also showed in vitro effect against SARS-CoV-2 [9] and in a RCT sponsored by the American National Institute of Allergy and Infectious Diseases [10]. The use of Remdesivir on hospitalized patients showed a significant reduction of length of stay from 15 (95% CI: 13-18) to 10 (95% CI: 9-11) days and suggests, also, a reduction in mortality of 27% (95% CI: -3% to 48%). Although a more recent RCT, sponsored by the World Health Organization (WHO), showed little or no impact on survival [11, 12], the drug has been authorized for treatment in UK, EU and the US, with a list price of <DOLLAR/>2340 for a 5-days treatment course [13, 14]. Dexamethasone, instead, is a broadly used corticosteroid with a low price of approximately 15<DOLLAR/> for a 10-days treatment course [15]. It was authorized by the Food and Drug administration in 1958 [16], and recently was recommended for use in Covid-19 patients with severe respiratory symptoms [17], as it has been showed that it reduces mortality by 36% (95% CI: 19% to 49%) for ventilated ICU patients and 18% (95% CI: 6% to 28%) for non-ventilated patients requiring oxygen [18].

At this stage the only published economic evaluation of Remdesivir is from the Institute for Clinical and Economic Review [19]. It estimates, from the health system perspective, the cost-effectiveness price benchmark of Remdesivir versus standard of care. It finds the price benchmark for Remdesivir to be cost effective for a population similar to the ACTT-1 trial [10], at a willingness to pay threshold of <DOLLAR/>50000 per quality adjusted life year (QALY) gained of <DOLLAR/>2470. In contrast, there are no published economic evaluations on the use of Dexamethasone for Covid-19 hospitalized patients.

The objective of this paper is to evaluate the cost utility of drug therapies for Covid-19. We do this through a cost utility decision tree model, using an health care prospective and one year horizon for an hypothetical cohort of Covid-19 hospitalized patients which represents the cohort of the Remdesivir clinical trial of [10]. We also test different alternative scenarios respect the base case, one when there is a mortality benefit for Remdesivir and one where hospital costs are taken into account in a different manner. We also compute the cost for life saved for Dexamethasone, as it is the only drug which has been proved to have a positive effect on mortality. Given the pandemic situation, resources should be directed to treatment and control of Covid-19, to save as much lives as possible. However, because of the rollout of effective vaccines [20], the pandemic is withdrawing in many part of the world [3], so the focus is shifting again on treatments for those who experience vaccine breakthrough infections and for those who cannot or won’t be vaccinated [21]. Therefore, an economic evaluation of the treatments should not be dismissed. Particularly, on Remdesivir doubts persist on its effectiveness respect to the trial results and the cost-effectiveness of the drug [22, 23]. Lately, beside the US and Europe even low-income countries have begun to use Remdesivir [24], sometimes even putting a ban on export [25]. Thus, because of the unproven effect on mortality this analysis will use utilities to compare the cost-effectiveness among treatments.

The remainder of the paper is structured as follows. “Method” section explains the model structure and the data used for the economic evaluations. In “Results” section we present the results of the analysis which serve as background for a more accurate discussion in “Discussion and concluding remarks”. “Discussion and concluding remarks” section ends the paper with some concluding remarks.

## Method

The study is a cost utility analysis based on a decision tree model developed with the R-package Heemod [26, 27]. Costs were considered from the perspective of the US health care services, thus consideration of societal costs is beyond the scope of this study. The use of Remdesivir, Dexamethasone or a hypothetical use of both drugs is compared to the alternative of standard care. The time horizon is one year so we take into account the utility for the survivors and the health care follow up costs.

### Model structure

**Fig. 1:**
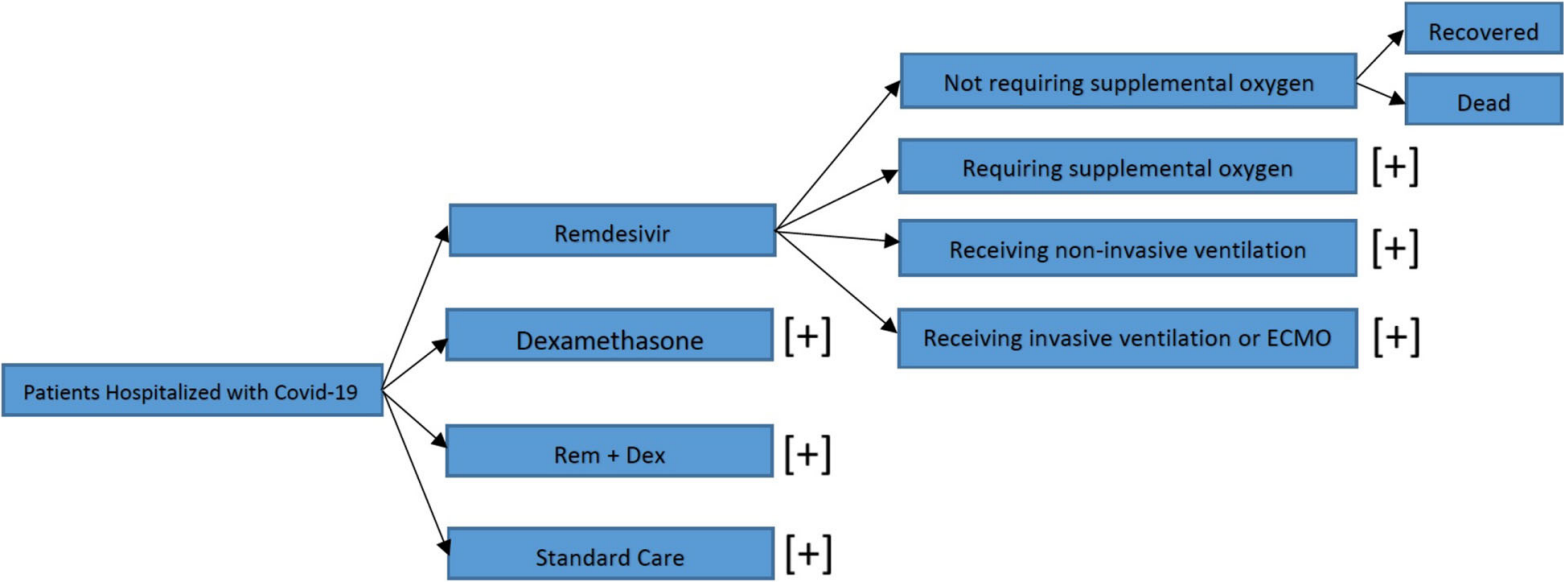
Decision Tree Schematic

The structure of the decision tree is shown in Fig. 1. We have 4 hypothetical cohort of 1000, 60 years old, Covid-19 hospitalized patients. One cohort is treated with Remdesivir, the second with Dexamethasone and the third with both drugs, while the control group receives standard care. The specification of the decision tree derives from a representation of the Remdesivir clinical trial [10].

All the patients, therefore, as enrolled in the trial, enter one of four status which represents the baseline severeness of illines indicated in the ordinal score of the RCT:

- Hospitalized, not requiring supplemental oxygen;
- Hospitalized, requiring supplemental oxygen;
- Hospitalized, receiving noninvasive ventilation or high-flow oxygen devices;
- Hospitalized, receiving invasive mechanical ventilation or ExtraCorporeal Membrane Oxygenation (ECMO).

Each patient admitted in one of the four categories described above would recover from the infection, and survive, or die, without any transition between the different degrees of hospitalization.

In the considered strategies, the patients receive the drugs as they are enrolled. The use of Remdesivir will have effect on the length of hospitalization resulting in a higher utility for the patients, and a decrease on the cost for the health care service.

We assume costs are divided equally in two parts: a fixed part that does not depend on the length of stay and a variable part correlated with the days of hospitalization. Of course, Remdesivir affects the latter.

There is no available information about whether Dexamethasone reduces the length of stay for the hospitalized patients. But, it has been demonstrated that Dexamethasone has an positive effect on the mortality for patients requiring external oxygen [18]. This positive effect affects the total utility gained as more patients survive. Moreover, there are no studies that examine the use of Dexamethasone together with Remdesivir.

In this paper, we assume that the effects of Remdesivir and Dexamethasone would add to each other: Remdesivir affects the hospital length of stay for all patients and Dexamethasone affects the mortality for patients requiring external oxygen, respectively. If a patient dies neither cost or utility is attached to the dead state, whilst the model takes into account the cost and utility of the patient while hospitalized even if she dies. In contrast, health care follow up costs are considered if she survives, as well as the utility for a year of healthy life. Since the dis-utilities of sequelae for Covid-19 hospitalization have not yet been estimated properly, they are non considered in this study. Moreover, there are not confirmed effects on the improved survival rate for Remdesivir patients. Thus, in our analysis we assume Remdesivir does not affect mortality rates. However, we run an additional base case scenario in which there is an effect as described in the meta analysis of [12].

We also run two more scenarios concerning the use of Remdesivir: one where costs of hospitalization are fixed and do not depend on the length of stay of patients, and another where hospital costs depend totally on the length of hospitalization.

### Model parameters

As for the specification of model parameters, information about data collection are as follows: - Data about the probability of being hospitalized in one of the four categories, the probability of outcome, the length of stay until recovery for both the control and the analyzed strategy cohorts are retrieved from the Remdesivir RCT [10]. - Data concerning Hazard Mortality Ratio (HMR) for the use of Dexamethasone in patient requiring external oxygen are instead retrieved from [18]. - Data about the Hazard mortality ratio for the Remdesivir’s scenario in which the anti viral has a positive effect on mortality are collected from the meta analysis of the World Health Organization (WHO) solidarity trial [12].

Costs of hospitalization, and follow up costs come from [28] and [29]. These studies estimate the costs of hospitalization in US particularly for Covid-19 patients, differentiating among the degree of the treatment (hospitalized, non-ventilated, and ventilated). Unfortunately, utilities for hospitalized Covid-19 patients have not been estimated yet, so we use as their proxies the utilities derived from pneumonia and influenza hospitalization, that are found in previous literature [30–32]. As base utility we use the mean utility of a 60 years old person in US [33]. All the model inputs and their references are reported in Table 1.

**Table 1:** Parameters of the model

Data Input Parameter	Value (range)	Distribution	Ref.
% of patients not requiring supplemental oxygen	13%	Point estimate	[10]
% of patients requiring supplemental oxygen	42%	Point estimate	[10]
% of patients receiving noninvasive ventilation	18.2%	Point estimate	[10]
% of patients receiving invasive ventilation or ECMO	26.8%	Point estimate	[10]
Prob. to die if hospitalized without supplemental oxygen	4.8% (1.6-14.3)	Beta(1.427,29.02)	[10]
Prob. to die if hospitalized with oxygen	12.7% (8.8-18.3)	Beta(21.05,143.41)	[10]
Prob. to die if receiving noninvasive ventilation	20.4%(13.7-29.8)	Beta(17.03,65.94)	[10]
Prob. to die if in ECMO	19.3% (13.8-26.5)	Beta(24.51,102.92)	[10]
Remdesivir Rate Ratio for time to recovery	1.29 (1.21-1.49)	LogN(0.255, 0.0735)	[10]
Remdesivir Hazard Mortality Ratio	0.91	Point Estimate	[12]
(only for the alternative scenario)			
Dexamethasone HMR for hospitalized with supplemental oxygen	0.82 (.72-.94)	LogN(-.198, 0.07)	[18]
Dexamethasone HMR for ventilated patients (invasive and non invasive)	0.64 (.51-.81)	LogN(-.446, 0.12)	[18]
Days of hospitalization without supplemental oxygen	6 (4-7)	Triangular	[10]
Days of hospitalization with supplemental oxygen	9 (7-10)	Triangular	[10]
Days of hospitalization receiving noninvasive ventilation	20 (14-26)	Triangular	[10]
Days of hospitalization receiving invasive ventilation or ECMO	28 (24-30)	Triangular	[10]
Cost of Remdesivir	<DOLLAR/>2340 (1755-2925)	Gamma(2340, 305)	[14]
Cost of Dexamethasone	<DOLLAR/>15	Point estimate	[15]
Total cost of hospitalization in standard care without supplemental oxygen	<DOLLAR/>9763 (7322 - 12203)	Gamma(9763, 1195)	[28]
Total cost of hospitalization in standard care with supplemental oxygen	<DOLLAR/>13767 (10325 - 17208)	Gamma(13767, 1711)	[28]
Total cost of hospitalization in standard care with non invasive ventilation	<DOLLAR/>34223 (25667 - 42778)	Gamma(34223,4113)	[28]
Total cost of hospitalization in standard care with invasive ventilation or ECMO	<DOLLAR/>61169 (45876 - 76461)	Gamma(61169,7403)	[28]
One year follow up costs for Covid-19 hospitalized survivors	<DOLLAR/>4132 (3099 - 5165)	Gamma(4132, 501)	[28]
Utility for hospitalized patients without supplemental oxygen	0.581(.472 -.729)	Beta(34.2, 23.97)	[30]
Utility for hospitalized patients with supplemental oxygen	0.5 (.4 -.6)	Beta(47.37, 47.37)	[30]
Utility for hospitalized patients with non invasive ventilation	0.23 (.18 -.23)	Beta(61.43, 207.97)	[31]
Utility for hospitalized patients with invasive ventilation or ECMO	0.05 (.02 -.08)	Beta(9.137, 171.78)	[32]
Base Utility	0.851	Point Estimate	[33]

Utilities and costs are attached to each node of the decision tree and to compute the their value for a individual hospitalized patient we proceed as follows:

- **Utilities**: for each kind of hospitalized patient we attach to the decision node a utility. This depends on the severeness of illness and consequently on the kind of hospitalization and, in addition, on the time spent in the ward. Without loss of generality, let us call $U^{SC}_{H}$ the utility given to a generic hospitalized patient in standard care (e.g.: patients not requiring supplemental oxygen), *D*_*H*_ the days spent in the ward, and *Q*_*D*_ the QALY of hospitalization. Then we have:
  1 $$ U^{SC}_{H} = D_{H} (Q_{H}/365)  $$
  If a patient receives the Remdesivir treatment, she has its length of hospitalization shortened by the Remdesivir rate ratio (RR). Thus, for patients receiving the anti viral treatment we have:
  2 $$ U_{H}^{Rem} = \frac{D_{H}}{RR}(Q_{H}/365)  $$
  Paradoxically, if we compute the utility in this manner we would have more utility for the no-Remdesivir arm with respect the Remdesivir’s one. Thus, we need to suppose that patients spend 30 days in this state, and so for Remdesivir patients we have:
  3 $$ U_{H}^{Rem} = \frac{D_{H}}{RR}(Q_{H}/365)+ \left(30-\frac{D_{H}}{RR}\right)(Q_{B}/365)  $$
  whilst for standard care patients we have:
  4 $$ U_{H}^{SC} = D_{H}(Q_{H}/365)+ (30-D_{H})(Q_{B}/365)  $$
  where *Q*_*B*_ is the base QALY for a 60 year old patient. Moreover, we use 30 days because in our analysis this time span is the longest time of hospitalization for any patient (see Table 1, Days of hospitalization receiving invasive ventilation or ECMO). Basically, each patient stays in the hospitalization state for 30 days. She can spend these 30 days as hospitalized (*D*_*H*_) with an attached utility of (*Q*_*H*_), or as not hospitalized (30−*DH*) with an attached utility of *Q*_*H*_. After 30 days, she moves to the next state, which is labelled as “recovered” if she survives, or as “dead” if she does not. Since the use of Dexamethasone does not influence the length of hospitalization, we have that the utility for the patients treated with Dexamethasone only is equal to the utility of standard care patients:
  5 $$ U_{H}^{Dex}=U_{H}^{SC}  $$
  whilst for patients receiving both Dexamethasone and Remdesivir the utility is equal to that of the Remdesivir patients:
  6 $$ U_{H}^{RemDex}=U_{H}^{Rem}  $$
  These utilities vary for all the four kinds of hospitalization. Each kind of hospitalization has a different *Q*_*H*_ and length of hospitalization *D*_*H*_, that would result in different *U*_*H*_. As a results, if a patient survives a year utility of healthy life *Q*_*B*_ is attached to the recovered node.
- **Costs**: for the hospitalization nodes we suppose that costs are divided equally in two parts, and from this assumption we can compute all costs for every kind of hospitalized patients in the four treatment strategies. First, without loss of generality, we take the total cost spent for a hospitalized patient in standard of care *Cost*_*SC*_ (e.g.total cost of hospitalization in standard care without supplemental oxygen), and divide it by two:
  7 $$ Cost_{{SC}}=Cost_{SC_{1}}+Cost_{SC_{2}}  $$
  with
  8 $$ Cost_{SC_{1}}=Cost_{SC_{2}}=\frac{Cost_{{SC}}}{2}  $$
  Thus, $Cost_{SC_{1}}$ is the fixed part
  9 $$ Cost_{SC_{1}}=Cost_{{Fix}}  $$
  and $Cost_{SC_{2}}$ the varying part, from which we compute the daily cost *Cost*_*Day*_:
  10 $$ Cost_{{Day}}=\frac{Cost_{SC_{2}}}{D_{H}}  $$
  The cost associated to a hospitalized patient in standard of care is:
  11 $$  Cost^{SC}_{{Tot}}= Cost_{{Fix}} + Cost_{{Day}}D_{H} = Cost_{{SC}}  $$
  To compute the cost associated to patients receiving Dexamethasone, say $Cost^{Dex}_{{Tot}}$, we just need to add the price of the treatment (*Cost*_*Dex*_) to Eq. 11 :
  12 $$ Cost^{Dex}_{{Tot}}= Cost_{{Dex}}+Cost_{{Fix}} + Cost_{{Day}}D_{H}  $$
  Patients receiving Remdesivir, however, have a shorter length of hospitalization. Thus, to compute the cost associated to patients receiving Remdesivir, say $Cost^{Rem}_{{Tot}}$, we need to divide *D*_*H*_ by the rate ratio *RR*, just as we did for utilities, in the varying part of the total cost, and next we have to add the price of the treatment:
  13 $$  Cost^{Rem}_{{Tot}}= Cost_{{Rem}}+Cost_{{Fix}} + Cost_{{Day}}\frac{D_{H}}{RR}  $$
  Finally, to compute the associated cost for patients that receive both treatments, we add the price of Dexamethasone to Eq. 13:
  14 $$  Cost^{RemDex}_{{Tot}}= Cost_{{Rem}}+Cost_{{Dex}}+Cost_{{Fix}} + Cost_{{Day}}\frac{D_{H}}{RR}  $$
  These costs vary depending on the severeness and on the length of the hospitalization. For example, in the base case scenario and for patients hospitalized not requiring supplemental oxygen we have *D*_*H*_=6 and *Cost*_*fix*_=<*DOLLAR*/>9763/2, and so on for all the other three kinds. Then, if the patient survives, a year for follow up costs is attached to the recovered state.

### Uncertainty analysis

One-way deterministic sensitivity analysis (DSA) is performed to examine the relative importance of the parameters respect to the standard of care for each of the three analyzed strategies. Thus probabilities, costs and utilities are varied by a lower and a higher value derived from the base case. These values are usually identified with respect to their 95% confidence interval, if available. Otherwise, the base case reference value is varied by ±25*%* from the point estimate. The low-high possible values allows us to determine the range of the Incremental Cost Effectiveness Ratio (ICER) due to parameter uncertainty, and to produce Tornado diagrams. Finally, a Probabilistic Sensitivity Analysis (PSA) with 10,000 Monte Carlo simulations is carried out in order to assess how changes of several parameters influence ICER. Simulations are done by choosing a random value from the distribution of the parameters. As suggested by [34], probabilities and utilities follow a beta distribution, whilst costs follow a gamma distribution. The reference distribution for each parameter can be found in Table 1. The results of the PSA are presented through the cost-effectiveness acceptability curves and in the incremental cost-effectiveness plane. Hereby, the probabilistic sensitivity analysis evaluates the level of confidence, i.e. the reliability, of the results of the analysis.

## Results

### Base case

**Fig. 2:**
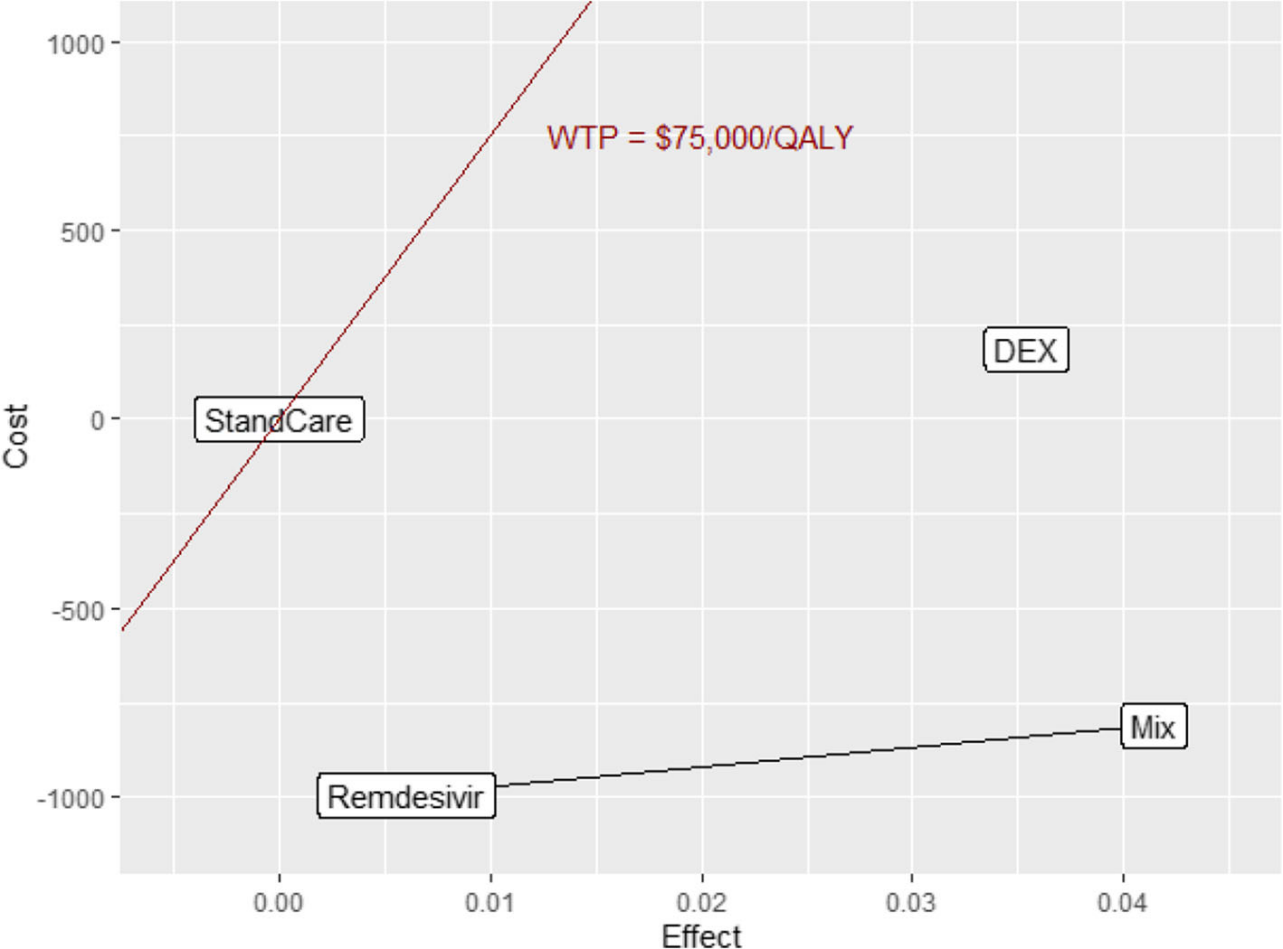
Base Case Cost-effectiveness plane

The base case analysis results are presented in Tables 2 and 3. Standard care for hospitalized Covid-19 patients has a cost of <DOLLAR/>33369.9 for 0.7673 QALY. The use of Dexamethasone for patients has an incremental cost effectiveness ratio of <DOLLAR/>5208/QALY versus standard care. The Remdesivir and the Remdesivir plus Dexamethasone (Rem + Dex) treatments both dominated the standard care treatment, as they were less costly and more effective. Table 3 shows how Dexamethasone and Rem+Dex do compare to Remdesivir alone. With respect to Remdesivir, the Dexamethasone treatment had an ICER of <DOLLAR/>40735.2/QALY, whilst the Rem + Dex treatment had an ICER of <DOLLAR/>5221.9/QALY with respect to the solo Remdesivir and it dominates the Dexamethasone treatment. Figure 2 summarizes these results in the incremental cost effectiveness plane. Hereby, we find that Remdesivir is the most cost saving strategy. This is due to the fact that Remdesivir would shorten the length of stay in hospital for patients, resulting in lower cost and a slight increase in utility. Dexamethasone, instead, is the most costly treatment, because it has no effect on the length of stay for the hospitalized patients but has a positive effect on the mortality. Moreover, since in this analysis we take into account the follow up cost for the survivors, the use of Dexamethasone leads to an increase of both cost and utility. The third treatment, which consists in the simultaneous use of Remdesivir and Dexamethasone, is the one that leads to the highest increase in utility and however is less costly than standard of care and Dexamethasone. This finding is probably due to our assumption that the effects of Remdesivir and Dexamethasone would add to each other, resulting in a shorter hospitalization thanks to Remdesivir and in a positive effect on mortality due to Dexamethasone.

**Table 2:** Base Case results (ref. Standard care)

Strategy	Cost	*Δ* Cost	QALY	*Δ* QALY	ICER
Standard Care	33369.9		0.7673		
Dexamethasone	33555.6	185.8	0.803	0.0357	5208
Remedesivir	32354.4	-1015.5	0.7734	0.0061	Dominant
Rem + Dex	32540.2	-829.71	0.809	0.0417	Dominant

**Table 3:** Base Case results (ref. Remdesivir)

Strategy	*Δ* Cost	*Δ* QALY	ICER
Remdesivir	ref.	ref.	
Dexamethasone	1201.3	0.0295	40735.2
Rem + Dex	185.8	0.0356	5221.9

#### Alternative Remdesivir scenarios

We also study the effect of Remdesivir depending on other assumptions which differ respect to our base model, and compute the cost for life saved for Dexamethasone. The results of this analysis are shown in Table 4.

**Table 4:** Base case results of the alternative scenarios

Strategy	Cost	*Δ* Cost	QALY	*Δ* QALY	ICER
Standard Care	33369.9		0.7673		
Remdesivir	32409.7	-960.2	0.7848	0.01748	Dominant
(Assuming mortality benefit)					
Remdesivir	35709.9	2340	0.7734	0.0061	384412.8
(Assuming only fixed costs)					
Remdesivir	28861.2	-4330.7	0.7734	0.0061	Dominant
(Assuming only cost per day)					
**Strategy**	**Cost**	***Δ* Cost**		**Life Saved**	**ICER**
Standard Care	29,686,258			ref.	
Dexamethasone	29,673,208	13050		42	313.79

In the first alternative scenario, we analyze the cost utility of Remdesivir in the case this drug has a positive effect on the patient mortality. So far the effect on mortality of Remdesivir has been disputed, therefore we run a scenario where Remdesivir has an hazard mortality ratio of 0.91 as found in the meta-analysis in [12]. Results show that the utility is almost three times greater because of the greater number of lives saved, but this would mean also higher costs due to the follow up of survived patients that cause this treatment to be not dominant respect to the standard of care. The other scenarios concern the assumption, of the base case model, that hospital costs are equally divided in two parts: a fixed part and a varying part that depends on the days of hospitalization. If we assume that hospital cost depends only on the fixed part, Remdesivir has an ICER of <DOLLAR/>384412.8/QALY, meaning that is not cost effective for any reasonable willingness to pay threshold. On the other hand, if costs depends on the days of hospitalization only, using Remdesivir would lead to a substantial saving in health care related costs. In the last possible scenario, we compute the cost for life saved for the Dexamethasone arm compared to standard care only, as Dexamethasone is the only treatment that has showed to have a positive effect on patient mortality. In this scenario, we do not consider the utility resulting from the length of staying hospitalized, neither the follow up costs for survivors. The results in Table 4 show a difference of <DOLLAR/>13050 for 1000 hospitalized patients, and 41.6 lives saved. This result leads to a cost for life saved of <DOLLAR/>315.79, well below any willingness to pay threshold.

### Sensitivity analysis

**Fig. 3:**
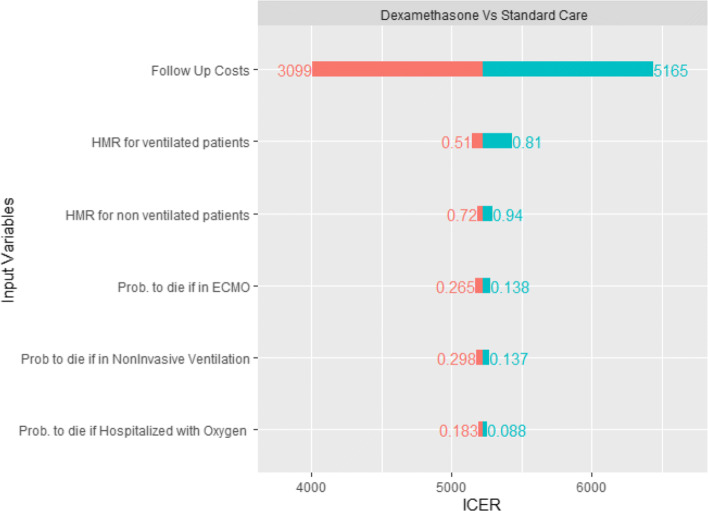
DSA Dexamethasone vs Standard Care

**Fig. 4:**
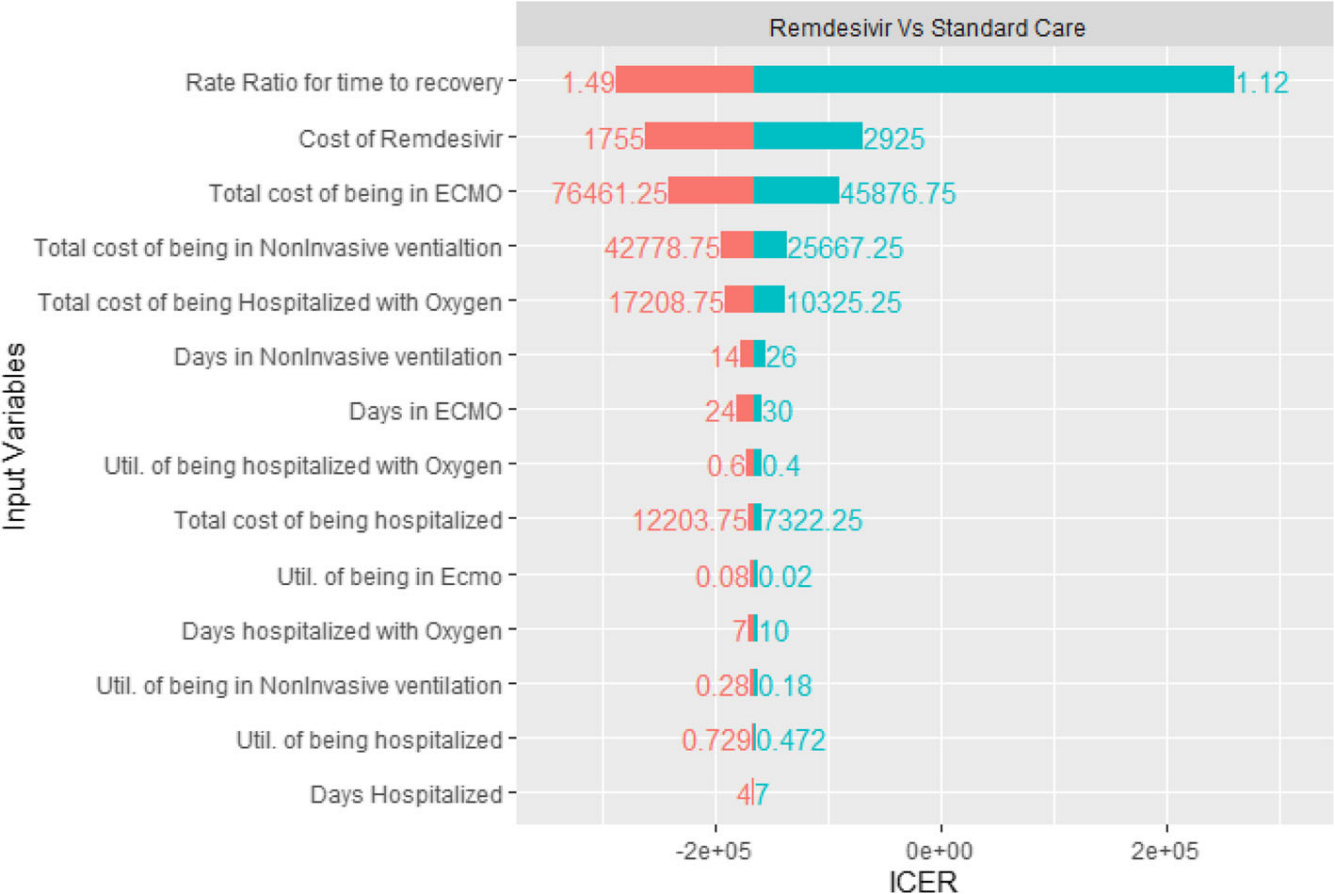
DSA Remdesivir vs Standard Care

**Fig. 5:**
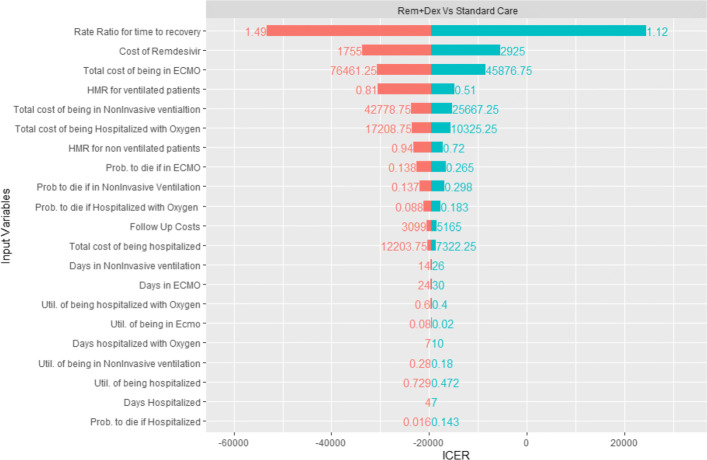
DSA Rem + Dex vs Standard Care

We run three one-way deterministic sensitivity analysis, one for each treatment (Dexamethasone, Remdesivir, and Remdesivir plus Dexamethasone) that is contrasted against the standard of care to check how model results are sensitive to parameter values. The results of these analysis are show in Figs. 3, 4 and 5 as tornado diagrams.

Results show that, for the Dexamethasone treatment, follow up costs are the most important parameter, but regardless the range of the variables the results are robust, as the maximum ICER is <DOLLAR/>6438 per QALY gained, and the use of Dexamethasone is still cost effective. The Remdesivir and the Rem+Dex treatments are still dominant respect to standard of care for any range of parameter, except when the Remdesivir rate ratio for time to recovery is in the lower bound of the confidence interval. In this case, when the rate ratio is 1.12, we have a maximum ICER of <DOLLAR/>24438/QALY for the Rem+Dex treatment, and a maximum ICER of <DOLLAR/>260614/QALY for the solo Remdesivir treatment. The results of the 10,000 Monte Carlo simulations are shown in Table 5. They do not differ from the base case scenario.

**Table 5:** PSA results (ref. Standard care)

Strategy	Cost	*Δ* Cost	QALY	*Δ* QALY	ICER
Standard Care	33371.7		0.7677		
Dexamethasone	33555.6	183.9	0.8028	0.0352	5229.1
Remedesivir	32388.4	-983.3	0.7736	0.0059	Dominant
Rem + Dex	32572.2	-799.4	0.8087	0.0411	Dominant

**Fig. 6:**
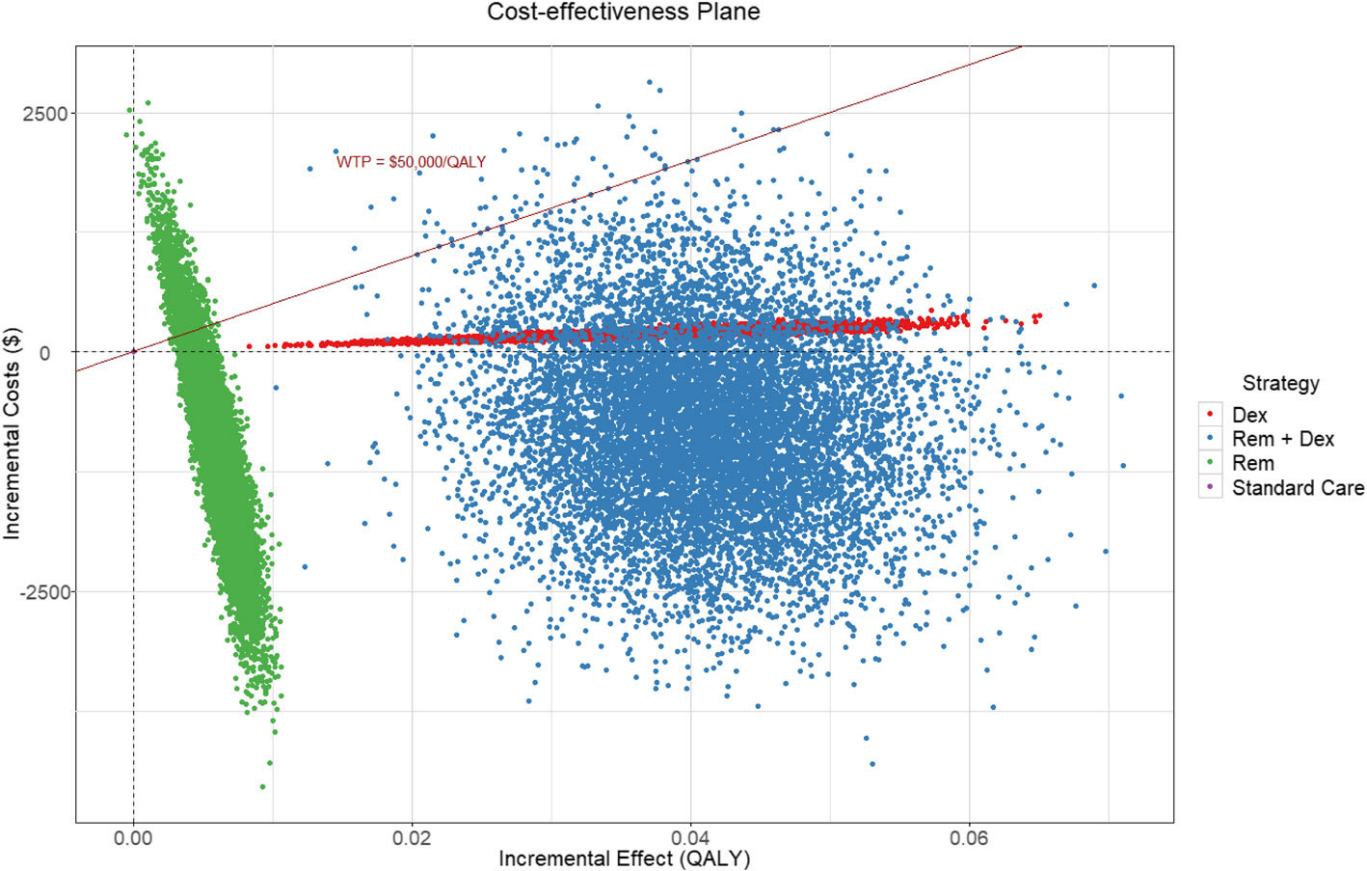
PSA Cost effectiveness plane

**Fig. 7:**
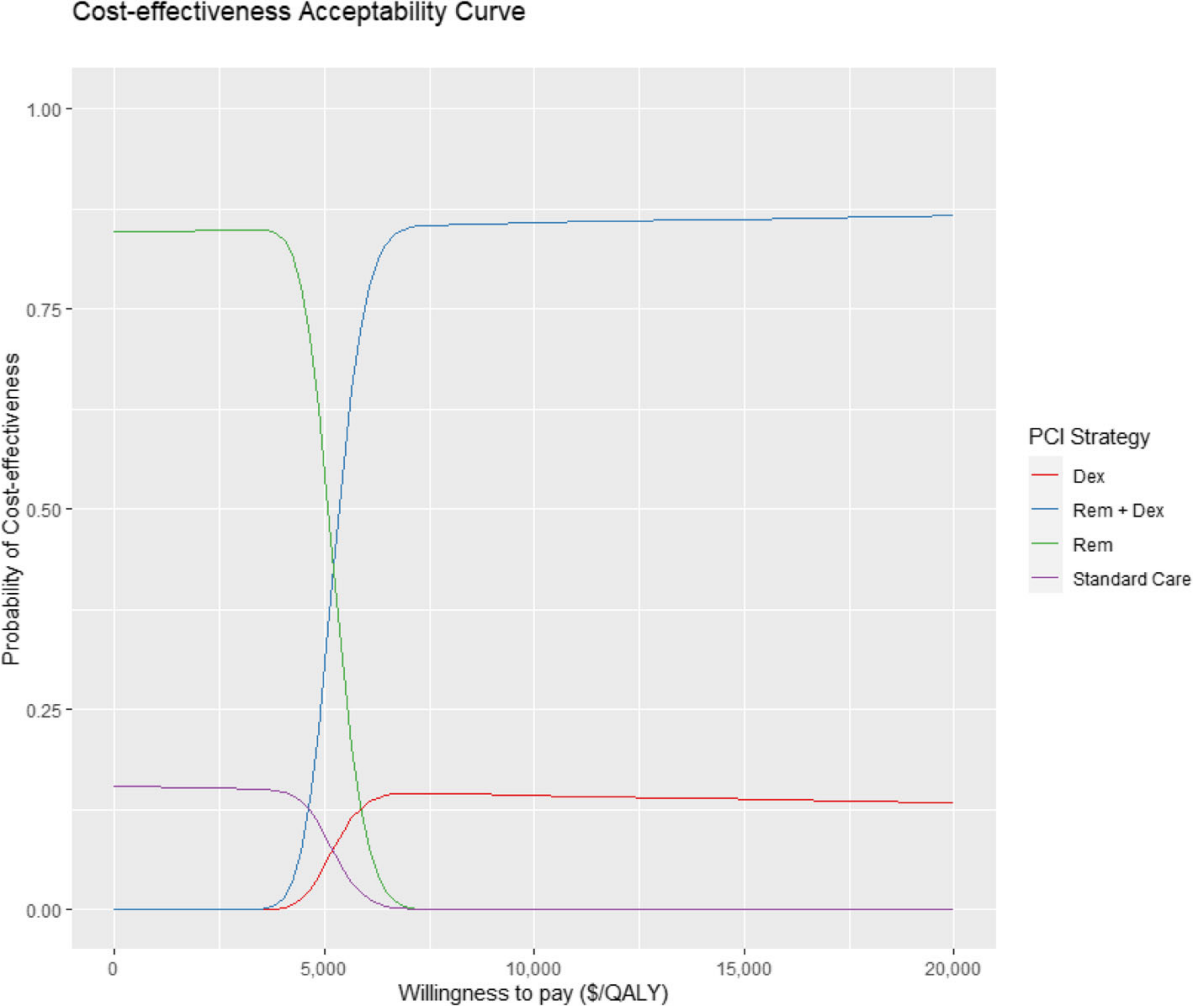
PSA Cost effectiveness acceptability curves

Moreover, we consider a representation of the simulation in the cost effectiveness plane and the willingness to pay threshold for <DOLLAR/>50000 in Fig. 6, and the acceptability curves for all the strategies in Fig. 7. From Fig. 6 we see that the majority of simulations for all the considered treatments lie below the <DOLLAR/>50000 WTP threshold. In particular, only the Remdesivir treatment has a significant part of the simulation outcomes located above the threshold line. In contrast, the Dexamethasone treatment is always below the threshold line. The cost effectiveness acceptability curve in Fig. 7 shows the probability of the interventions being cost effective under different WTP thresholds. For example, for a WTP below <DOLLAR/>5000 the most cost-effective treatment would be Remdesvir, but as the threshold increases the Rem+Dex treatment would be more likely to be favoured.

## Discussion and concluding remarks

In the base case analysis, we found the Remdesivir is the most cost saving treatment. This could be counter intuitive given the drug high price, but if we assume that the health costs are related to the length of hospitalization (and they probably are to some extend) we can see why it is cost saving. Another point in favour of the use of Remdesvir is the fact that hospital beds in many countries are a scarce resource, and when the pandemic surges they rapidly get full [35]. Thus, the use of a drug that allows us to vacate hospital beds and human resources should be suggested, even if it does not have effect on mortality. In contrast, Dexamethasone is not cost saving respect to standard of care, although is a low cost treatment. This finding is due to the fact that, for the health care perspective considered in this study, we take into account up to a year of follow-up costs for the survivors, which are in a greater number respect to Remdesvir and standard of care treatments, and also because from the clinical trials there are no sign of reduction for the length of hospitalization. As stated in “Model structure” section, there are not yet findings from randomized control trials on the use of Remdesivir together with Dexamethasone but, if the effects of the two treatments would add each other, this analysis shows that this combined treatment would lead to a substantial saving in health care costs and in the greatest increase in utility for patients respect to standard of care, as it results as the most favorable strategy as shown by the acceptability curves represented in Fig. 7.

Although this study analyzes the cost utility of Covid-19 treatments from a United States prospective, as costs and utility are estimated from studies on this country, we might argue that results are still robust for other developed countries such as Canada, UK or the EU. However, because the main cost driver is hospitalization, for developing countries where hospitalization costs are much lower than the US or other G8 countries, the use of Remdesivir for a price of <DOLLAR/>2430 could be problematic, and depending on the WTP threshold results may lead to favour Dexamethasone.

There are several limitations to our analysis. The cost utility analysis is based on very limited literature on the effectiveness of the treatments analyzed in this model. Moreover, the use of Dexamethasone and Remdesivir taken together has not been proved to work in any randomized control trial, but we think this joint treatment should be analyzed anyway as there is a specific trial [36] currently in progress. Hence, given that Covid-19 is fairly a new disease, new information could become available and the model assumptions could change. Moreover, as we tried to mimic as best as possible the Remdesivir RCT, this analysis did not take into account potential disease progression over time, nor the difference in age, comorbidities, sex and ethnicity among the patient cohort, nor the potential adverse events in terms of nosocomial infection that could be associated with a prolonged hospital stay [37, 38]. Also, the effect of Remdesivir for reduction of days of hospitalization is still a disputed issue, as there have been mixed results in other RCTs as, for example, the WHO solidarity trial [12]. In the latter study, however, the trial has not been designed to primarily test the time to recovery [39]. Moreover, other observational studies have shown the benefits of Remdesivir even for ventilated patients [40, 41]. Next, hospital costs where assumed to be related at some extend to the patients length of stay. We do not know how much length of stay influences the total cost of hospitalization, and to account for this uncertainty we also considered the two alternative simulation scenarios. Given the burden on mortality for the pandemic, we also compute the cost for life saved for the Dexamethasone arm, which has shown to be very convenient, and below any WTP threshold.

Last but not least, data concerning utility for Covid-19 has not been estimated yet, so we had to rely on data derived from influenza and pneumonia hospitalization. Finally, we have assumed that after the discharge from hospital there would not be any impact on mortality or utility and, consequently, there are concerns on effects of Covid-19 post infection [42]. These are yet to be determined, hence they are not considered in this analysis, For these reasons, the time horizon of the model is only one year long, but we are aware that a different model time span could cause important changes in the outcomes [43]. If we had computed the cost and utility for a longer time horizon (e.g. lifetime horizon) we would have taken into account the baseline mortality risk, that would differ substantially with the cohort patient’s age. Moreover, we should have been looking at the decrease on baseline QALY [33] and the increase in the healthcare cost that survived patients would face in their remaining life.

In conclusion, any of the treatments analyzed in this study resulted to be cost effective for the willingness to pay threshold of developed countries. Caveats apply on the use of Remdesivir as its effectiveness depends strongly on the hospital costs. Moreover, its effect is still unknown when it is used concurrently with Dexamethasone, so additional information is required on this topic. Further research about economic evaluations is needed, as more treatments for Covid-19 patients are being introduced, such as monoclonal antibodies, and the treatments guidelines are continually updated [44, 45]. Anyway, this analysis supports the idea that the use of Remdesivir and/or Dexamethasone is effective from an economic standpoint as it has been shown that it allows to save costs and lives.

## Data Availability

The datasets used and/or analyzed during the current study are available from the corresponding author on reasonable request.

## Abbreviations

COVID-19: COrona VIrus Disease 2019
Sars-Cov-2: Severe acute respiratory syndrome coronavirus 2
RCT: Randomized Control Trials
WHO: World Health Organization
ICU: Intensive care unit
QALY: Quality adjusted life year
ECMO: ExtraCorporeal Membrane Oxygenation
HMR: Hazard Mortality Ratio
RR: Rate Ratio
DSA: Deterministic sensitivity analysis
ICER: Incremental Cost Effectiveness Ratio
PSA: Probabilistic Sensitivity Analysis
WTP: Willingness to pay
